# The Unfolded Protein Response Pathway in the Yeast *Kluyveromyces lactis*. A Comparative View among Yeast Species

**DOI:** 10.3390/cells7080106

**Published:** 2018-08-14

**Authors:** Mariana Hernández-Elvira, Francisco Torres-Quiroz, Abril Escamilla-Ayala, Eunice Domínguez-Martin, Ricardo Escalante, Laura Kawasaki, Laura Ongay-Larios, Roberto Coria

**Affiliations:** 1 Departamento de Genética Molecular, Instituto de Fisiología Celular, Universidad Nacional Autónoma de México, 04510 Mexico City, Mexico; melvira@email.ifc.unam.mx (M.H.-E.); edominguez@email.ifc.unam.mx (E.D.-M.); lkawasak@ifc.unam.mx (L.K.); 2 Departamento de Bioquímica y Biología Estructural, Instituto de Fisiología Celular, Universidad Nacional Autónoma de México, 04510 Mexico City, Mexico; ftq@ifc.unam.mx; 3 Laboratory for Membrane Trafficking, VIB-KU Leuven Center for Brain & Disease Research and Department of Neurosciences, KU Leuven, 3000 Leuven, Belgium; abril.escamillaayala@kuleuven.vib.be; 4 Instituto de Investigaciones Biomédicas “Alberto Sols” (CSIC-UAM), Arturo Duperier 4, 28029 Madrid, Spain; rescalante@iib.uam.es; 5 Unidad de Biología Molecular, Instituto de Fisiología Celular, Universidad Nacional Autónoma de México, 04510 Mexico City, Mexico; longay@ifc.unam.mx

**Keywords:** yeast, endoplasmic reticulum, stress, UPR, Ire1, Hac1, Kar2

## Abstract

Eukaryotic cells have evolved signalling pathways that allow adaptation to harmful conditions that disrupt endoplasmic reticulum (ER) homeostasis. When the function of the ER is compromised in a condition known as ER stress, the cell triggers the unfolded protein response (UPR) in order to restore ER homeostasis. Accumulation of misfolded proteins due to stress conditions activates the UPR pathway. In mammalian cells, the UPR is composed of three branches, each containing an ER sensor (PERK, ATF6 and IRE1). However, in yeast species, the only sensor present is the inositol-requiring enzyme Ire1. To cope with unfolded protein accumulation, Ire1 triggers either a transcriptional response mediated by a transcriptional factor that belongs to the bZIP transcription factor family or an mRNA degradation process. In this review, we address the current knowledge of the UPR pathway in several yeast species: *Saccharomyces cerevisiae*, *Schizosaccharomyces pombe*, *Candida glabrata*, *Cryptococcus neoformans,* and *Candida albicans*. We also include unpublished data on the UPR pathway of the budding yeast *Kluyveromyces lactis*. We describe the basic components of the UPR pathway along with similarities and differences in the UPR mechanism that are present in these yeast species.

## 1. Introduction

Secreted and transmembrane proteins are synthesised in ribosomes that are attached to the endoplasmic reticulum (ER) membrane and are co-translationally processed and folded in the lumen of this organelle. Protein folding is accomplished by chemical modification, including the addition of oligosaccharides (*N*-glycosylation) and formation of disulphide bonds. These modifications are essential for the proper function of the proteins. Certain physiological, environmental, or pathogenic conditions can cause the accumulation of unfolded proteins, generating a condition known as ER stress. Experimentally, ER stress is induced by treatment with agents that either inhibit *N*-glycosylation, such as tunicamycin (Tn) or 2-deoxyglucose (2-DOG), or agents that disrupt the formation of disulphide bonds such as dithiothreitol (DTT) and β-mercaptoethanol (β-ME). In order to respond to these harmful conditions, eukaryotic organisms activate a conserved signalling pathway termed the unfolded protein response (UPR). Many components and mechanisms of this pathway are conserved between species and they are dedicated to restoring ER homeostasis by increasing protein folding capacity and by decreasing the load of new proteins arriving to the ER. In metazoans, the UPR has three branches, each one named after the protein involved in sensing the ER stress stimulus. They are known as IRE1 (inositol-requiring enzyme 1), PERK (protein kinase RNA-like endoplasmic reticulum kinase) and ATF6 (activating transcription factor 6). The presence of these branches is species-dependent; however, the most conserved component of the UPR is the IRE1 branch, which is present in all eukaryotes including yeasts and the social amoeba *Dictyostelium discoideum* [1,2].

The IRE1 branch is composed by Ire1, that acts as a sensor of the ER stress. Under ER stress, Ire1 processes the mRNA of a bZIP transcription factor (in most cases known as Hac1). Once synthesised, the transcription factor is transported into the nucleus where it regulates transcription of a variety of genes such as those that encode chaperones, post-translational modification enzymes and proteins for proteolytic degradation. This pathway was initially described in *Saccharomyces cerevisiae*, and it was later detected in other yeast species, but it is not universally conserved among yeasts. For instance, the fission yeast *Schizosaccharomyces pombe* lacks a Hac1 orthologue and the UPR does not consist of a transcriptional response; instead, Ire1 triggers an ER-targeted mRNAs decay pathway. In this review, we describe the current knowledge regarding the UPR in several yeast species, namely *S. cerevisiae*, *S. pombe*, *Candida glabrata*, *Cryptoccocus neoformans* and *Candida albicans*. In the third section, we present original data (obtained in our group) regarding the UPR in *Kluyveromyces lactis*. To our knowledge, this is the first review that describes the UPR of these six yeast species and we expect that it will be of great interest to researchers in the field. 

## 2. The UPR in *Saccharomyces cerevisiae*

**Figure 1:**
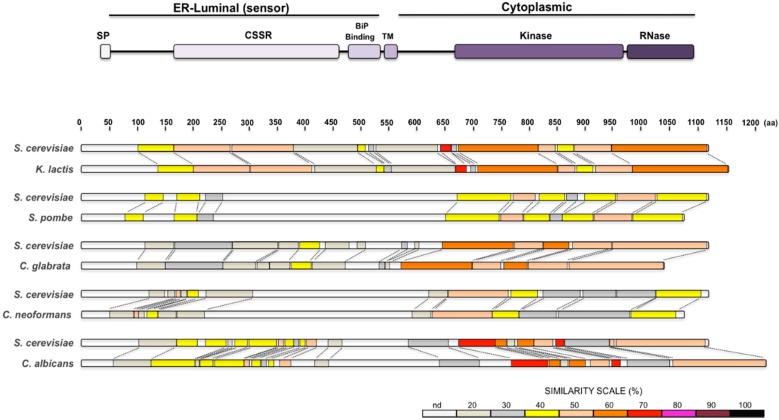
(**Top**) Diagram of the Ire1 protein structure. Signal Peptide (SP), Core Stress Sensing Region (CSSR), BiP Binding domain, Transmembrane segment (TM), and the Kinase and RNase domains were deduced from the *S. cerevisiae* Ire1 protein. (**Bottom**) Pairwise alignments between the *S. cerevisiae*, *K. lactis*, *S. pombe*, *C. glabrata*, *C. neoformans* and *C. albicans*, Ire1 orthologues. The Ire1 protein of each species is compared to the *S. cerevisiae* one. Protein sequences were analysed with SIM local alignment tool and results were visualized with LALNVIEW. Protein schemes are drawn to scale.

The IRE1 pathway was initially described in *S. cerevisiae*. In this system the *Sc*Ire1 protein has been extensively characterised. In this work we do not intend to describe in detail all the information accumulated regarding *Sc*Ire1, due to space limitations; we will simply refer to the general characteristics that are relevant to this review. *Sc*Ire1 is a 1115 amino acid residue transmembrane protein (Figure 1). This protein has a sensor domain at its N-terminus that lies inside the lumen and interacts with unfolded proteins, while its C-terminus lies in the cytosol and contains a serine-threonine kinase domain and an endoribonuclease domain [3,4]. A single transmembrane segment that lies in a region with very low similarity to the Ire1 of other yeast species connects the luminal N-terminal sensor domain with the cytoplasmic kinase and endoribonuclease domains (Figure 1). The activity of *Sc*Ire1 is regulated by the ER-luminal resident chaperone *Sc*Kar2 (BiP) (see below). Briefly, *Sc*Kar2 is bound to the N-terminus of *Sc*Ire1 under normal conditions and dissociates in response to ER stress [5,6]. Dissociation of *Sc*Kar2 leads to *Sc*Ire1 dimerisation and ultimately to its oligomerisation by binding to unfolded proteins through its core stress-sensing region (CSSR) located in the sensor domain [7,8,9]. The crystal structure of the *S. cerevisiae* CSSR suggests that it is able to form a groove similar to that present in the histocompatibility complex where the unfolded proteins may be captured [7]. The interaction with unfolded proteins may be essential for *Sc*Ire1 activation [10].

*Sc*Ire1 was initially isolated as essential for growth in the absence of inositol [11]. It was later determined that the null mutant was lethal in the presence of ER stress inducers [12]. An *S. cerevisiae* mutant lacking Ire1 is auxotrophic for inositol because it is unable to induce adequate expression of Ino1, the inositol-1-phosphate synthase that constitutes the rate-limiting step in the *de novo* inositol biosynthesis pathway [11,13]. The absence of inositol induces the UPR in wild-type yeast cells, and treatment with Tn induces *INO1* expression in an *Sc*Ire1-dependent manner [14]. *Sc*Ire1 is not required for cell growth in normal conditions but it is essential for growth in the presence of ER stress inducers such as Tn and β-ME [14,15]. 

Mutations that affect either the groove where unfolded peptides bind (M^229^A, F^285^A, Y^301^A) or those that affect oligomerisation and clustering (F^247^A, W^426^A) affect the response to ER stress-inducing agents [9,10]. Additionally, mutations that substitute K^702^ and N^1057^ abolish kinase and RNase activities respectively and also affect growth in ER stress inducers [3,16,17,18]. These data indicate that in *S. cerevisiae* all three Ire1 activities, i.e., the unfolded protein sensor, the kinase activity and the RNase activity, are essential for Ire1 function in the UPR pathway and required for proper ER stress response. 

**Figure 2:**
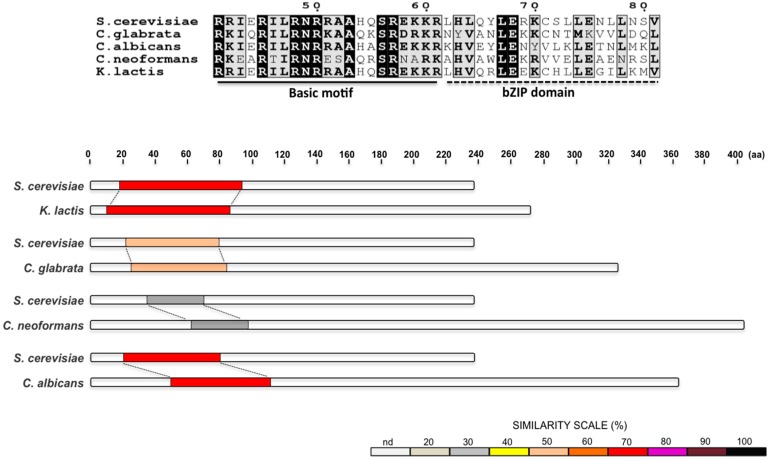
(**Top**) Alignment of the basic motif and the bZIP domain of Hac1 (or Hxl1) of yeast species. Amino acids fully conserved are depicted in black boxes. Amino acid coordinates correspond to the *S. cerevisiae* protein. (**Bottom**) Pairwise alignments between the *S. cerevisiae*, *K. lactis*, *S. pombe*, *C. glabrata*, *C. neoformans* and *C. albicans*, Ire1 orthologues. The Ire1 protein of each species is compared to the *S. cerevisiae* one. Protein sequences were analyzed with SIM local alignment tool and results were visualized with LALNVIEW.

Once *Sc*Ire1 is active, it, in turn, activates the transcription factor Hac1p, which comprises 238 amino acids and belongs to the basic-leucine zipper (bZIP) family. It contains a basic DNA binding region of 21 amino acids followed by a leucine zipper motif of 20 amino acids (Figure 2) [19]. In the presence of ER stress, *Sc*Hac1 binds as a homodimer to promoters of UPR targets [19,20] in sequences known as UPRE (unfolded protein response element) motifs. It has been proposed that *Sc*Hac1 binds to long and short UPREs (see below) through at least two different mechanisms, allowing a wide regulation of UPR gene transcription [20]. Conserved N and R residues within the basic DNA binding region of *Sc*Hac1 are essential for recognition of palindromic or semi-palindromic UPRE DNA target sites (Figure 2). These residues make direct contact with the major groove of DNA [21,22]. A lack of Hac1 does not compromise cell growth in normal conditions but it does cause sensitivity to ER-stress-inducing drugs such as Tn and DTT, and also to caffeine [23], and as in the case of an *Sc*Ire1 null mutant, a lack of *Sc*Hac1 causes inositol auxotrophy [13,24].

**Figure 3:**
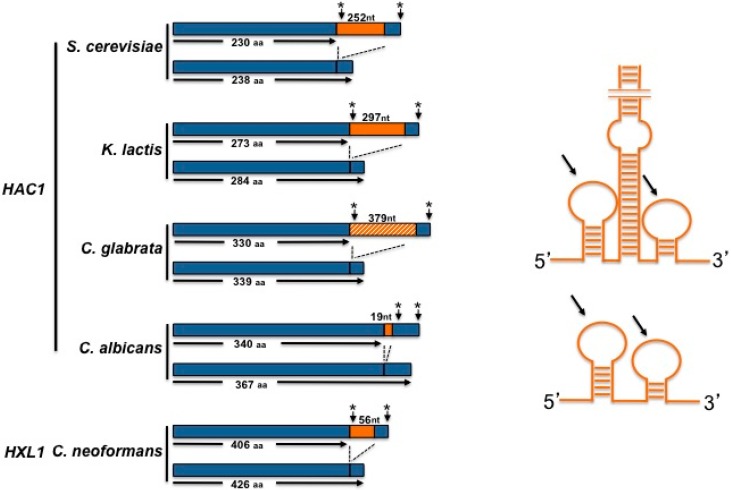
(**Left**) Scheme of un-spliced and spliced *HAC1* (or *HXL1*) RNAs. Sizes of putative protein products from un-spliced and spliced RNAs are depicted. Introns (and their sizes in nucleotides) are shown by the orange boxes. The putative *HAC1* intron of *C. glabrata*, that appears not to be processed is indicated by the slashed box. Stop codons are indicated by asterisk. (**Right**) Scheme of putative stem-loop structures of large and small introns. Arrows indicate 5′ and 3′ exon-intron boundaries. Structures are not drawn to scale.

Although the *ScHAC1* precursor mRNA (*HAC1*^u^, for uninduced *HAC1*) is constitutively produced, the *Sc*Hac1 protein is not detected [25]. *ScHAC1* mRNA contains an unconventional 252 nucleotide intron near its 3′ end (Figure 3) that is specifically processed only by the RNase domain of *Sc*Ire1 [4,26,27]. *ScHAC1* pre-mRNA contains a translational attenuator that is located near the 5′ end of the intron [26]. The attenuation of translation is exerted by base pairing between the intron and the 5’ untranslated region, forming a loop structure where the ribosomes are stalled [28,29] (see the analogous mechanism of *K. lactis* attenuation depicted in Section 3). After splicing, the two exons are joined together by the RNA ligase Rlg1, leading to the formation of a mature mRNA (*HAC1^i^*, for induced *HAC1*) [13]. This splicing allows the substitution of the last 10 amino acid residues of *Sc*Hac1^u^ by 18 residues, rendering a protein of 238 amino acids (*Sc*Hac1^i^) [25,27]. The novel 18 amino acids at the C-terminus tail of Hac1 function as the transcription transactivation domain [30]. *ScHAC1* mRNA splicing by *Sc*Ire1 is a highly selective and efficient process that is promoted by the binding of pre-mRNA to a docking site formed by a positively charged motif located in the cytosolic linker domain of Ire1. The R^647^ and R^650^ residues located within this basic motif appear to play an important role in the binding of *HAC1* pre-mRNA [17]. Additionally, *ScHAC1* mRNA forms a stem-loop structure at its 3’ UTR that contains an important component, the 3′ UTR bipartite element (3′BE), which consists of two juxtaposed short motifs located at the distal end of the loop. This stem structure cooperates with the intron stalling structure to target the *ScHAC1* mRNA to the Ire1 oligomer foci in order to allow efficient splicing of the mRNA. The two short motifs are highly conserved in several yeast *HAC1* orthologues [29].

Dissection of promoter sequences in the *ScKAR2* gene allows the identification of a 22-bp unfolded protein response element [31,32] to which *Sc*Hac1 binds directly [19,25]. Within the 22-bp element a central semi-palindromic core of 7 bp (5′-CAGNGTG-3′) known as UPRE-1 motif, is essential for the transcriptional activity of *ScKAR2* under ER stress [19]. The UPRE-1 motif, later determined to comprise from 11 to 13 bp [20], is present in several genes that are UPR targets [33,34]. Genes that are *Sc*Hac1 targets lacking the UPRE-1 motif contain a distinct sequence called UPRE-2 [35]. This sequence is 6 to 7 bp long (5′-TACGTGT-3′) and binds *Sc*Hac1 with high affinity [20].

*Sc*Kar2 is an ER-resident chaperone that belongs to the Hsp70 family. It has an ATPase domain near its N-terminus adjacent to the substrate-binding domain [36]. ATPase activity is required for its participation in the translocation of proteins across the ER membrane [37]. *Sc*Kar2 also participates in ER-associated protein degradation by maintaining luminal substrates in a retrotranslocation-competent state [38]. *Sc*Kar2 negatively regulates the UPR through its interaction with *Sc*Ire1. Under ER stress conditions, binding of unfolded proteins to *Sc*Kar2 induces its dissociation from *Sc*Ire1, leading to the activation of the UPR pathway [6,39]. *Sc*Kar2 is an essential and abundant protein, the synthesis of which is further induced by the presence of unfolded proteins [32,40].

## 3. The UPR in *Kluyveromyces lactis*

*K. lactis* is a biotechnologically important yeast which diverged before the genome duplication that gave rise to the *Saccharomyces* species [41]; thus, it represents a suitable organism for making evolutionary comparisons with *S. cerevisiae*. 

The *K. lactis* genome encodes orthologue proteins of the IRE1 branch of the UPR signalling pathway, namely Ire1, Hac1, and Kar2. The KLLA0D13266g ORF of *K. lactis* has been identified as putative homologue of the *IRE1* gene of *S. cerevisiae*. It comprises 3459 nucleotides including the stop codon and codes for a protein of 1152 amino acids. Comparative analysis shows that the putative protein has 48% identity with *Sc*Ire1, and exhibits a structure similar to that of *S. cerevisiae* (Figure 1). From the sequence analysis of *Kl*Ire1, a luminal dimerisation domain at the N-terminal region, a serine/threonine protein kinase catalytic domain, and an endoribonuclease domain located at the C-terminus can be predicted. *Kl*Ire1 conserves the K^737^ and N^1096^ residues, which are essential for the kinase and RNase activities in *S. cerevisiae*. Additionally, *Kl*Ire1 conserves the R residues (673 and 676) that in *S. cerevisiae* participate in the binding of *HAC1* RNA. 

**Figure 4:**
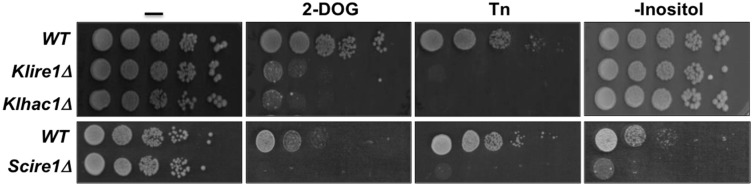
Effect of *IRE1* and *HAC1* inactivation on the *K. lactis* growth properties. *K. lactis* null mutants were obtained by standard homologous recombination introducing in both cases the *URA3* selective cassette. *K. lactis* cells were grown in YPD (1% yeast extract, 2% peptone, and 2% Glucose) until 0.5 OD_600_ then were washed and suspended in fresh YPD. Cells were spotted as 10 fold serial dilutions on YPD plates containing 15 mM 2-DOG or 50 ng/mL Tn and on plates of 2% glucose, 0.5% ammonium sulfate, 0.17% yeast nitrogen base w/o amino acids and w/o inositol. *S. cerevisiae* wild type and *ire1∆* strains were included as controls. Cells were plated on the same media except that the 2-DOG concentration was 20 mM and the Tn concentration was 500 ng/mL. Plates were incubated at 30 °C and photographed 48 h later.

Experimental data from our group showed that *Kl*Ire1 is involved in the UPR pathway in *K. lactis*; a *Klire1* mutant is sensitive to ER stress induced by Tn or 2-DOG (Figure 4). However, unlike *S. cerevisiae*, in *K. lactis IRE1* is not essential for growth in the absence of inositol (Figure 4), indicating that in this species the Ire1 function may differ, or that *K. lactis* may synthesise inositol in the absence of *Kl*Ire1. 

**Figure 5:**
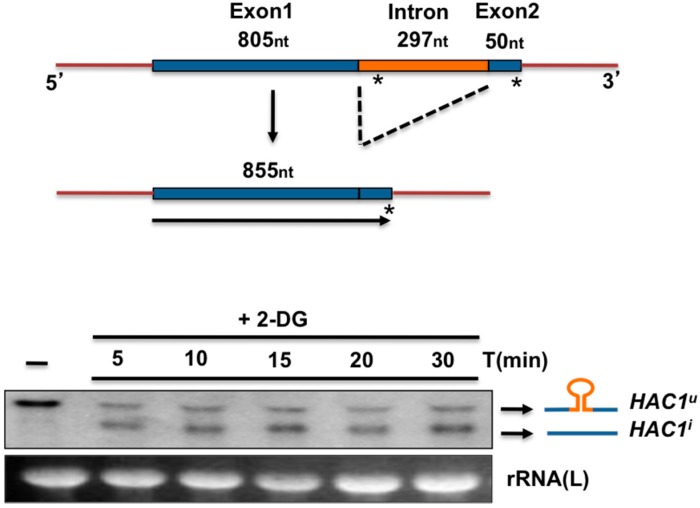
(**Top**) Diagram of the precursor and processed forms of *K. lactis HAC1* RNA. Exon and intron sizes (nucleotides) were determined by sequence analysis. Size of the induced *HAC1* (*HAC1*^i^) includes the stop codon. (**Bottom**) Northern blot analysis of *HAC1* processing. *K. lactis* cells were grown in YPD (1% yeast extract, 2% peptone, and 2% Glucose) until 0.5 OD_600_. Cells were treated with 20 mM 2-DOG for the indicated times. Total RNA was extracted with the conventional Trizol protocol. Boiled RNA was loaded in a 1% agarose gel, electrophoresed, and transferred to a nylon membrane. The RNA was probed with a fragment containing the first 500 nt of the *HAC1* ORF labelled with ^32^P and visualized in a phosphorimager. The large rRNA was used as a load control.

A *K. lactis HAC1* gene has been identified in the *K. lactis* genome database. *KlHAC1* is a 1152 nucleotide gene that encodes a pre-mRNA formed by two exons of 805 and 50 nucleotides respectively and a long intron of 297 nucleotides (Figure 3), which has been identified as characteristic in several *Saccharomyces* species and some other yeasts [42]. Transcription of the *KlHAC1* gene produces an mRNA that is processed upon ER stress induction. After 30 min of exposure of *K. lactis* cells to 2-DOG, the presence of two forms of the transcript are evident (Figure 5), the larger form corresponds to the unprocessed *KlHAC1* mRNA (*HAC1*^u^ mRNA), and the second to the mature form (*HAC1*^i^ mRNA). In *K. lactis*, the sequence of the *HAC1*^i^ mRNA predicts a 284 amino acids protein that has the features of a bZIP transcriptional factor; namely, a conserved DNA binding domain and a basic leucine zipper domain (Figure 2).

**Figure 6:**
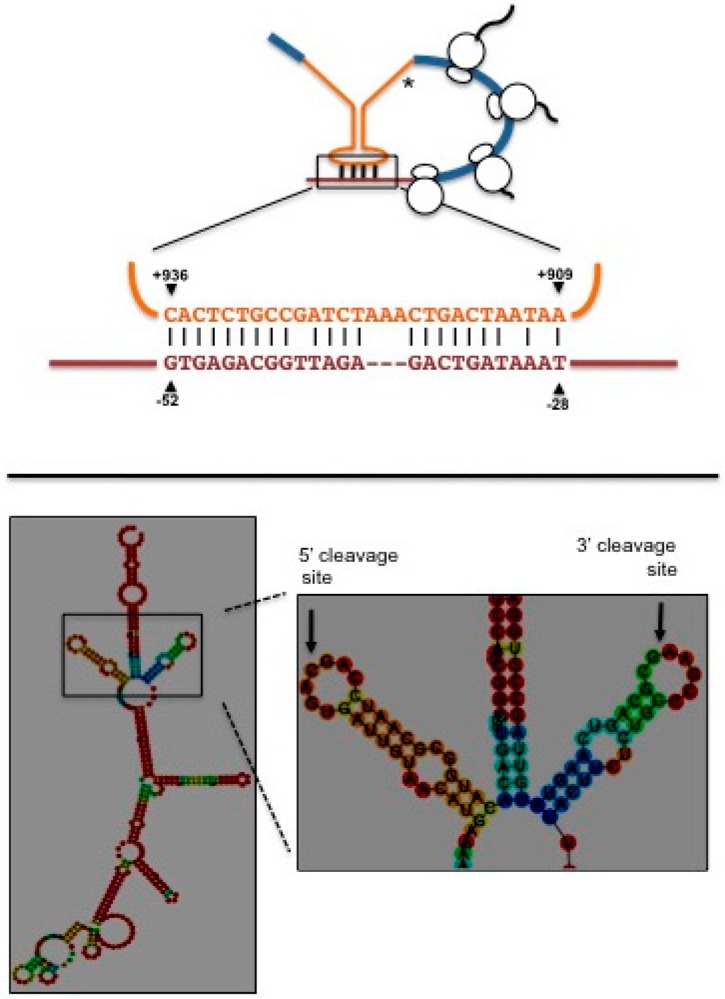
(**Top**) Diagram of a predicted translation attenuation structure formed in the *K. lactis HAC1* pre-mRNA. Exons are indicated in blue, intron is indicated in orange and 5′ untranslated region in brown. Coordinates are relative to the first nucleotide of the translation start codon. Asterisk indicates an in-frame stop codon located within the intron. (**Bottom**) Predicted stem-loop secondary structure of the exon-intron boundaries of *K. lactis HAC1* pre-mRNA. Arrows pinpoint the 5′ and 3′ cleavage sites. The predicted secondary structure was obtained using the RNAfold web server from the ViennaRNA Web Services.

Sequence analysis of both the *KlHAC1*^u^ and *KlHAC1*^i^ revealed that the pre-mRNA contains conserved cleavage motifs at the 5′ and 3′ intron-exon boundaries. Secondary structure analysis showed that these regions are capable of folding in a stem-loop structure (Figure 6). This sort of structure together with the 5′ and 3′ motifs is essential for the recognition and further cleavage of the pre-mRNA. We also found that there is a region in the intron (+909 to +936) that can base-pair with a region in the 5’UTR (−52 to −28) of the mRNA (Figure 6), allowing the formation of a translation attenuation structure like that of the *S. cerevisiae* mRNA and other *HAC1* RNAs with long introns [42,43]. Another interesting feature of the *K. lactis HAC1* mRNA is the presence of a conserved bipartite element, located in the 3′UTR, which in *S. cerevisiae* appears to be important in targeting the mRNA to Ire1 oligomers [29]. 

The aforementioned characteristics suggest that *K. lactis HAC1* mRNA is regulated and activated in a similar manner as that of *S. cerevisiae*, and that its translation may be modulated by the regulatory intron which upon ER stress is cleaved by the endonuclease activity of *Kl*Ire1 at the 5’ and 3’ specific splicing sites, and the exons are joined. This would render the *KlHAC1 mRNA* translatable, and the active transcriptional factor Hac1 would be produced.

Like *K*lIre1, *Kl*Hac1 is also a key element in the ER stress response pathway of *K. lactis*. A null mutant of *KlHAC1* is sensitive to agents that induce ER stress, such as Tn or 2-DOG (Figure 4). Additionally, as in the case of *KlIRE1*, this gene is dispensable for growth in the absence of inositol (Figure 4).

The *K. lactis KAR2* gene codes for the major ER chaperone, Kar2. The gene was identified by its high similarity with the *S. cerevisiae* gene. It has 2040 nucleotides and predicts a 679 amino acid protein, with 77.3% identity with the *S. cerevisiae* Kar2 [44].

*Kl*Kar2 displays characteristics typical of the Hsp70 type of chaperones such as a hydrophobic leader sequence, and an ER retention signal at its C-terminus, which is important in order to prevent its secretion. In *K. lactis* the retention signal of Kar2 is the tetrapeptide DDLE, while in *S. cerevisiae* it is HDEL; this difference seems to be important for the specificity of the retention system [44,45]. The *K. lactis* Kar2 sequence contains a potential *N*-glycosylation site at the C-terminal region, although it is not known whether it is functional in this budding yeast [45]. Additionally, the *Kl*Kar2 C-terminus shows some sequence conservation with that of *S. pombe* and cross-reacts with an antibody raised against the BiP of this latter species [46].

**Figure 7:**
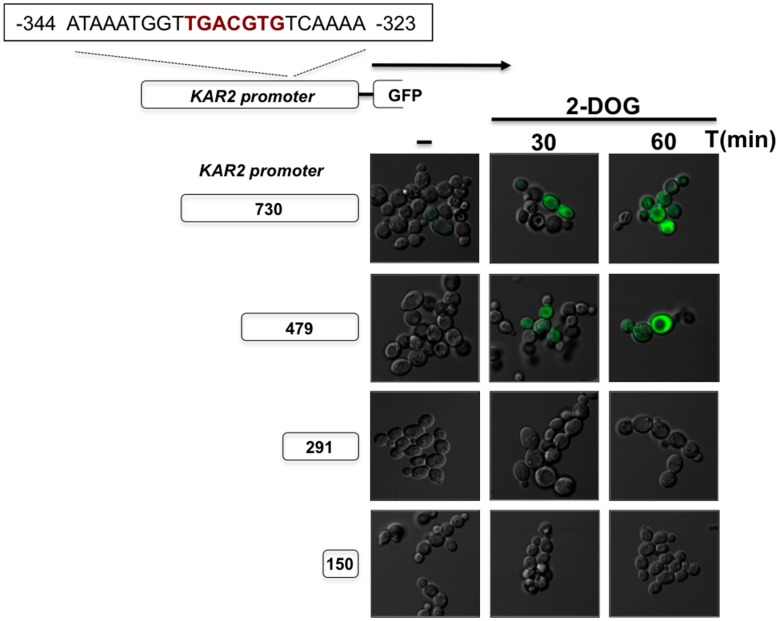
Detection of *K. lactis KAR2* promoter activity upon ER stress. The putative *KlKAR2* promoter (730 nucleotides of the upstream start codon region) and the indicated serial deletions fragments were fused to the *GFP* reporter gene. Constructions were cloned in an episomal multicopy *K. lactis* plasmid and used to transfect wild type cells. Cells were grown in YPD until 0.5 OD_600_ and treated with 2-DOG for the indicated times. Cells were visualized under epi-fluorescence microscopy and photographed.

**Figure 8:**
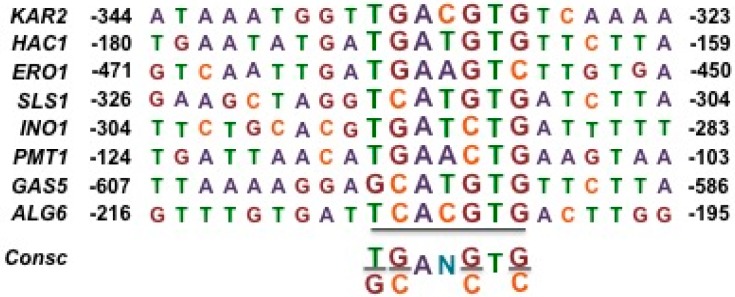
Blast search of the putative *K. lactis* UPRE motif (shown in brown in Figure 7) in putative ER stress-responsive genes. The search was directed to the indicated 5′ untranslated regions of the indicated genes. Coordinates are relative to the first nucleotide of the respective translation start codon.

We found that the *K. lactis KAR*2 gene is upregulated under ER stress. Treatment of *K. lactis* cells with 2-DOG induced over-expression of a GFP reporter gene fused to the *KAR2* promoter (Figure 7). By deletion analyses, we also identified a 211 nucleotide region with promoter activity that responded to 2-DOG induction (from −291 to −479). Within this region, we detected a 22 nucleotide motif (Figure 7) with some identity to the *S. cerevisiae* UPRE [20,31]. Furthermore, inside this motif the heptanucleotide TGACGTG was detected, which exhibits high similarity to UPRE-1 of *S. cerevisiae*. This motif was searched in promoter regions of several *K. lactis* genes that in *S. cerevisiae* have been identified as UPR-responsive genes. This analysis allowed us to identify the consensus sequence T/GG/CANG/CTG/C which may represent the *K. lactis* UPRE responsive element (Figure 8).

## 4. The UPR in *Schizosaccharomyces pombe*

*S. pombe*, the fission yeast, is a model organism used to analyse different aspects of cellular physiology. Interestingly, this yeast species lacks *HAC1* and the ER stress response is mediated by an Ire1-dependent mRNA degradation.

The Ire1 of *S. pombe* has the same structural features as *Sc*Ire1 (Figure 1). It has a luminal domain and a cytosolic portion that contains the kinase and endoribonuclease domains. The full length *Sp*Ire1 sequence shows only 24% identity with *Sc*Ire1, which indicates a wide evolutionary distance between the two proteins. *Sp*Ire1 contains the K^682^ and N^1014^ residues, which have been shown to be essential for kinase and RNase activities in *S. cerevisiae* respectively [3,16,17]. However, the R residues that may form the basic motif of the putative RNA docking site [17,18], and that are well conserved in other yeast species are not present in *Sp*Ire1, suggesting that it might not bind a specific RNA molecule.

*S. pombe* Ire1 null mutants are sensitive to ER stress inducers such as DTT and Tn [47] and since *S. pombe* is a natural auxotroph for inositol due to the absence of the inositol-1-phosphate synthase, the lack of inositol in the medium is lethal to *S. pombe* [48,49]. In this yeast, inositol is essential for mating and sporulation [50]. Although the mechanism remains unknown, it has been proposed that the effect of inositol may be indirect, possibly through regulation of membrane and cell wall composition [51]. 

Unlike *S. cerevisiae* and other yeast species, no specific substrate has been identified for *Sp*Ire1, suggesting that *S. pombe* lacks a Hac1 orthologue. In contrast to *S. cerevisiae* and other yeast species the UPR in *S. pombe* is not mediated by a transcriptional reprogramming; instead, it has been observed that the *Sp*Ire1 RNase activity is involved in a mechanism termed Regulated Ire-Dependent Decay (RIDD) [52]. This process was first described in *Drosophila* [53] but it has also been observed in plants [54] and mammalian cells [55,56]. In *S. pombe* upon ER-stress, *Sp*Ire1 cleaves ER-localised mRNAs at consensus sites, leading to free 5′ and 3′ end-containing mRNA fragments that are rapidly degraded by exoribonucleases in a 5′-3′ direction [57] and by the exosome in the 3′-5′ direction [53], thus alleviating the ER-protein load. The *Sp*Ire1 mRNA substrates contain a consensus UG/CU sequence flanking the cleavage sites. These cleavage sites reside within the coding sequences of mRNAs, resulting in the stalling of ribosomes engaged in translation. Those ribosomes are then liberated by a ribosome/mRNA decay pathway known as ‘no-go decay’, which consists of the endonucleolytic cleavage of the RNA and the Dom34/Hbs1-dependent recycling of the ribosome [58]. 

Approximately 31% of the mRNAs regulated by *Sp*Ire1 code for proteins involved in lipid metabolism, and particularly in sterol metabolism. Sterol has also been shown to induce ER stress and to trigger UPR in *S. cerevisiae* [59]. Despite the fact that it is not known how the reduction in sterol synthesis regulates the toxicity associated with ER stress, it has been suggested that this kind of stress affects vesicular sterol transport and that therefore, when sterol biosynthesis is reduced, ER membrane fluidity may be stabilised [52].

One of the RIDD substrates is the mRNA that codes for the Grp78 chaperone orthologue BiP1 [52,60]. *Sp*Ire1 cleaves the *BiP1* mRNA within the consensus UG/CU sequence located in its 3′-UTR, leading to the loss of the poliA tail. Paradoxically, *Sp*Ire1 activity does not increase the *SpBiP1* mRNA degradation rate; instead, it makes it more stable, possibly due to the elimination of an RNA degradation sequence without affecting its translation efficiency. 

In *S. pombe*, Bip1 is a 663 amino acid protein essential for cell viability and its expression is induced by various stresses, such as heat shock and Tn. It contains an ER retention signal [60] and a predicted *N*-glycosylation site [46]. Indeed, a small portion (10%) of *Sp*BiP1 is rapidly N-glycosylated upon synthesis and this glycosylation does not change with time. As one might expect, Tn prevents the appearance of the glycosylated form of BiP1 [46]. Although the synthesis of the mRNA of *Sp*BiP1 is not increased upon ER stress, its extended stability after SpIre1-dependent cleavage following ER stress results in an increase of the *Sp*Bip1 protein [52]. Finally, it appears that elimination of the 3′-UTR mRNA processing site in *Sp*BiP1 induces a significant impairment in the response to ER stress conditions [52].

## 5. The UPR in *Candida glabrata*

*C. glabrata* is one of the most common human pathogenic yeasts. It is phylogenetically related to *S. cerevisiae*, and it has the canonical Ire1 kinase and the transcriptional factor Hac1. However, the ER stress response mechanism in this yeast is very different; particularly, *CgHAC1* mRNA is not spliced by *Cg*Ire1, and *Cg*Ire1 regulates the response in an Hac1-independent manner.

*C glabrata* Ire1 is a 1036 amino acid protein that conserves the typical Ire1 domains: the N-terminal hydrophobic signal sequence, an ER luminal domain, a transmembrane segment, a serine/threonine kinase domain and a nuclease domain. Overall, *Cg*Ire1 displays 49% similarity and 33% identity with *Sc*Ire1 (Figure 1). *Cg*Ire1 is required for cellular response to ER stress inducers such as Tn and DTT, and this function requires its kinase and the ribonuclease activities [61]. However, the ribonuclease activity of *Cg*Ire1 does not seem to be required for the *CgHAC1* RNA splicing (see below); instead it appears to participate in the degradation of ER-associated mRNAs through a RIDD pathway similar to that of *S. pombe* [61]. Accordingly, *Cg*Ire1 does not trigger a transcriptional response to ER stress; instead, transcription is regulated by the calcium signalling pathway which depends on calcineurin phosphatase [61] (see below).

*C. glabrata* has a single *HAC1* orthologue containing a highly conserved bZIP domain and a conserved DNA binding region [61] (Figure 2). Overall, the *Cg*Hac1 transcription factor shows low similarity and low identity with *Sc*Hac1. The *CgHAC1* pre-mRNA contains a predicted intron of 379 nucleotides (Figure 3), which may potentially form a stem-loop structure, but it apparently lacks the consensus Ire1 splicing recognition sequences [61]. Unlike *Cg*Ire1, a lack of *Cg*Hac1 does not induce sensitivity to ER stress inducers and *Cg*Hac1 remains un-spliced in both stressed and non-stressed conditions. A lack of *HAC1* pre-mRNA splicing under ER stress conditions supports the notion that in *C. glabrata* there is a RIDD pathway that is *Cg*Ire1-dependent but *Cg*Hac1-independent [62]. Nevertheless, un-spliced *Cg*Hac1 is able to induce transcription of UPR genes in *S. cerevisiae*, indicating that it has conserved its structure and function [61]. 

The transcriptional response to ER stress in *C. glabrata* depends on calcineurin signalling and on the Slt2 MAPK pathway [61]. In this yeast, calcineurin prevents cell death upon ER stress by regulating calcium influx through the Crz1 transcription factor [61]. The calcineurin-Crz1 pathway is also required for the response of *C. glabrata* to various stress stimuli and for virulence [63]. The Stl2 MAPK may also exert an ER stress surveillance function to ensure transmission of healthy ER to daughter cells, as it does it in *S. cerevisia*e [62,64]. In *C. glabrata*, the gene transcription program triggered by ER stress appears to be more closely related to calcineurin-Crz1 regulated genes than to increasing the folding capacity of the ER [61,65]. In fact the *CgKAR2* promoter lacks a consensus UPRE sequence and its expression depends on the Crz1 transcription factor [61]. In summary, *C. glabrata* monitors ER stress by means of three pathways acting in parallel: the Ire1-RIDD pathway, the calcineurin-Crz1 pathway and the Stl2-surveillance pathway. *Cg*Ire1 is also required for virulence, although its role in the infectious process remains unknown [61]. 

## 6. The UPR in *Cryptococcus neoformans*

*C. neoformans* is a basidiomycetous yeast. It is the most common cause of severe pulmonary infections and meningoencephalitis in immunocompromised patients. This yeast has an unfolded protein response pathway involved in ER stress response and virulence [66,67,68].

*C. neoformans* has a conserved Ire1 protein. Overall *Cn*Ire1 displays 25% identity with *Sc*Ire1. It contains the typical Ire1 domains, a sensor-luminal domain, a Ser/Thr protein kinase, and a ribonuclease domain (Figure 1). It also contains the catalytic sites (Lys^641^ and Arg^1010^) for kinase and ribonuclease activities. A lack of Ire1 in *C. neoformans* generates sensitivity to ER stress inducers like Tn and DTT, and the *ire1* mutant also shows a variety of pleiotropic effects such as thermosensitivity and sensitivity to cell wall damaging agents [69]. The mutant lacking *Cn*Ire1 is avirulent since it is defective in forming the antiphagocytic capsule that is essential for evading the host immune response [69,70]. Inositol is required for mating and virulence and *C. neoformans* can use myo-inositol as a sole carbon source [71]; but there is no report that *Cn*Ire1 is required for its synthesis.

*Cn*Ire1 processes the *HXL1* pre-mRNA (the *HAC1* orthologue), which encodes a bZIP transcription factor that appears to be phylogenetically distant from *Sc*Hac1 (Figure 2). Hxl1 shows the lowest sequence conservation on the basic DNA binding domain compared to the other bZIP factors described in this work (Figure 2). However, like other *HACs*, *HXL1* contains an unconventional intron of 56 nucleotides whose splicing sites are well conserved with *basidiomycetes* and *ascomycetes* fungi (Figure 3) [70]. Although a small proportion of spliced *HXL1* mRNA coexists with the unspliced molecule in unstressed conditions, most pre-RNA is spliced by *Cn*Ire1 under ER stress conditions [69]. In contrast to *S. cerevisiae* and *K. lactis*, the *CnHXL1* intron does not seem to contain sequences complementary to the 5′-UTR region, disregarding an attenuation mechanism for negative regulation of translation. This suggests that the unspliced mRNA can be translated, yielding a 406 amino acid protein in contrast to the 426 amino acids of the induced Hxl1 protein [70]. In *C. neoformans*, an alternative mechanism of post-transcriptional regulation has been described. Splicing and stability of the *HXL1* mRNA is regulated through binding of Puf4, a component of the pumilio-FBF family of mRNA binding proteins that facilitates splicing under ER-stress conditions and attenuates mRNA degradation during ER stress attenuation [72]. 

A Kar2/BiP chaperone has been identified in *C. neoformans*, whose expression under ER stress conditions is regulated by the Ire1-Hxl1 pathway [69,73]. *Cn*Kar2 is an essential protein with 679 amino acid residues that displays an Hsp70 domain and an ER retention signal [73,74]. *Cn*Kar2 is required for cellular response to ER stress and high temperature, and for maintenance of cell wall integrity [70,73].

Aside from the ER stress response, *Cn*Ire1 has other functions. It also participates in the biosynthesis of the antiphagocytic capsule, in thermotolerance, in azole drug resistance, partially in the genotoxic stress response and in the maintenance of cell wall integrity [69,70]. Except for capsule production and thermotolerance these functions are at least partially mediated by Hxl1, while capsule biosynthesis seems to be dependent only on *Cn*Ire1 [70]. Furthermore, *Cn*Ire1 has a role in the sexual mating and unisexual differentiation of *C. neoformans*. While sexual mating is dependent on the activity of *Cn*Kar2, the same-sex mating is independent [68,74]. Both, opposite- and uni-sexual reproductions are independent of the Hxl1 transcription factor [68]. This indicates that *C. neoformans* has evolved unique features of the UPR pathway that are not present in other eukaryotic organisms.

## 7. The UPR in *Candida albicans*

*C. albicans* is an opportunistic human fungal pathogen. The yeast-mycelia morphological transitions play an important role in its pathogenesis.

*C. albicans* contains a typical Ire1 protein. Its full length comprises 1224 amino acid residues. By primary sequence comparison *Ca*Ire1 contains the common structural domains, i.e., the sensor-luminal domain, the protein kinase domain and the ribonuclease domain (Figure 1). Overall CaIre1 displays 45% similarity and 31% identity with *Sc*Ire1. Like other yeasts, *Ca*Ire1 conserves the amino acid residues involved in kinase and RNase activities. Although, no direct evidence of the involvement of *Ca*Ire1 in the ER unfolded protein response has been obtained, its structural features indicate that it may activate a bZIP transcription factor (see below). It also appears that defective *Ca*Ire1 mutants display high sensitivity to cell wall stress inducers such as caspofungin [75] and show defective filamentation that alters their pathogenic capacity [76]. *C. albicans* is able to synthesise inositol *de novo* and can take it up from the media; however, *Ca*Ire1 has not been shown to participate in any of these processes [77]. 

In *C. albicans* the most studied component of the UPR pathway is the transcription factor Hac1. This protein displays high sequence similarity within the putative DNA-binding region to other yeast Hac1 proteins (Figure 2). Under ER stress conditions, the *CaHAC1* mRNA is processed analogously to the *S. cerevisiae HAC1* [78]. In contrast to other yeast species, however, the *CaHAC1* intron is only 19 bp long and it is located near the 3′ end of the precursor mRNA (Figure 3), although it is apparently capable of forming the stem-loop structure characteristic of this kind of introns. Processing of the intron results in the synthesis of a transcription factor with a novel 27 C-terminus [78] that may form the transactivation domain. The sequence at the intron/exon boundaries in *Ca*Hac1 are well conserved [78], but the unspliced form of *Ca*Hac1 is unable to complement a *Schac1∆* mutant, indicating that *Sc*Ire1 is unable to process the *CaHAC1* intron [78]. However, the fact that the spliced form of *CaHAC1* is able to complement the *S. cerevisiae* mutant indicates that *Ca*Hac1 is the functional homologue of *Sc*Hac1 [78]. Due to the small size of the *CaHAC1* intron, a translation attenuation mechanism similar to that present in *S. cerevisiae* and *K. lactis*, seems to be discarded.

CaHac1 is required in order to trigger a cellular response to ER stress inducers such as Tn and DTT. In fact, it appears that under ER stress, *Ca*Hac1 triggers expression of a group of genes involved in secretion, cell wall biogenesis and vesicle transport among other processes [78].

Like in *C. glabrata*, the ER stress response in *C. albicans* is dependent on the calcineurin-CRZ1 pathway [79]. Nevertheless, it seems that in this species, the calcineurin pathway is required as an assisting mechanism to regulate the Hac1-dependent UPR genes [79]. Additionally, *C. albicans* cells that are defective in calcineurin and Crz1 activities are highly sensitive to Tn and DTT [80].

*C. albicans* expresses a Kar2 chaperone orthologue, which is essential for cell survival [81] but nothing is known regarding its participation in the ER stress response pathway. It has been determined that *Ca*Kar2 can partially complement a lack of Kar2 in *S. cerevisiae*, alleviating the thermosensitivity displayed by the *Sckar2∆* mutant. Additionally, *Ca*Kar2 displays *in vitro* protein translocation activity, suggesting that it may participate in the secretory pathway of *C. albicans* [81].

## 8. Concluding Remarks 

**Figure 9:**
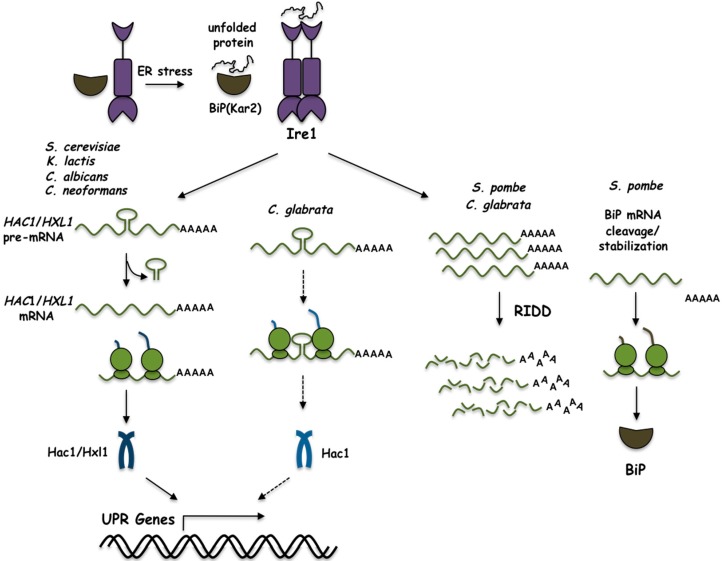
The unfolded protein response pathway in several yeast species. The accumulation of unfolded proteins in the ER leads to the activation of Ire1. In *Sc*., *Kl*., *Ca.*, and *Cn*., Ire1 splices the *HAC1*/*HXL1* pre-mRNA leading to its translation. The synthesised bZIP factor (Hac1/Hxl1), regulates transcription of UPR-responsive genes. In *Sp*., and *Cg*., Ire1 cleaves ER-localised mRNAs through the Regulated Ire-Dependent Decay (RIDD) pathway. In *Sp*., Ire1 also cleaves the BiP mRNA in the 3′ UTR region leading to its stabilization and translation. As described in the text, the unspliced *HAC1* RNA of *Cg* may induce transcription of UPR-responsive genes in *S. cerevisiae* (depicted by the dotted arrows), although there is no evidence that this process can actually occur in *C. glabrata*.

The unfolded protein response (UPR) is a signalling pathway that is activated in response to ER stress to restore and maintain ER homeostasis. In mammalian cells, it is composed by three branches: IRE1, PERK, and ATF6. IRE1 is the most conserved branch of the UPR. It is preserved in all eukaryotes and is the only branch present in yeast species. Even though the Ire1 sensor is highly conserved, the mechanisms to handle the ER stress response varies in different species. The kinase and RNase domains of Ire1 proteins are greatly similar among different species. In particular, the catalytic residues of those domains are highly conserved in all eukaryotes [82]. Upon ER stress, ER homeostasis can be restored in two ways: by increasing the protein folding capacity, and by decreasing the load of proteins arriving to the ER. The folding capacity may be increased through transcriptional upregulation, while the reduction of the protein load can be achieved by selective decay of ER-localised mRNAs. In any case, it seems that the RNase activity of Ire1 has a crucial role in the mechanism of the UPR, either degrading a large set of mRNAs or processing a very specific mRNA to produce a transcriptional response. In mammalian cells, both mechanisms are present, and are triggered depending on cellular conditions and cell type [52,53,56]. However, in yeast species the strategy is different; some yeasts rely on a transcriptional response to regulate genes needed to cope with unfolded protein accumulation, while others initiate a selective decay of ER-localised mRNAs (Figure 9). In *S. cerevisiae, K. lactis, C. albicans* and *C. neoformans* Ire1 processes the pre-mRNA of a bZIP transcription factor, Hac1 (in *Sc.*, *Kl.*, and *Ca.*) or Hxl1 (in *Cn*.); while in *S. pombe* and *C. glabrata* the response is mediated by Ire1 through degradation of ER targeted-mRNAs by the RIDD pathway (Figure 9).

It has been proposed that the RNase activity used in the RIDD process represents the ancestral mechanism, since it is less specific with a broader mRNA degradation capacity. Evolution of this process implied loss of this broad degradation capacity and the acquisition of a more specialised mechanism to splice a specific target [52]. Through this last mechanism Ire1 removes an unconventional intron; interestingly, it has been shown that the sequence of the Hac1/Hxl1 intron is conserved only at the splice recognition sites, while its size and structure vary among different species; it is very short in metazoan, filamentous fungi, and several yeasts, whereas some other yeasts have long introns [83]. The long intron of *S. cerevisiae* is shared by some closely related species, and it is suggested that the translation attenuation mechanism that depends on the 5′ UTR described in *S. cerevisiae* is present only in this kind of long introns [42]. We have found that *K. lactis* presents a long intron containing a sequence complementary to the 5′ UTR, which is similar to that of *S. cerevisiae*, therefore the attenuation mechanism could be conserved in *K. lactis*. Some yeasts lack the regulatory intron or even have lost the ortholog of the bZIP mRNA and therefore have evolved other mechanisms for the induction of the UPR. These different strategies indicate that yeast species have optimized the mechanisms of their UPR to adapt to their specific lifestyles. The UPR pathways of the yeast species described in this review show intriguing differences that may expand our understanding of their phylogenetic relationships and the utility of the mechanisms present in each organism to deal with and adapt to their particular niche.
